# Identification of Prognosis-Related Genes in Bladder Cancer Microenvironment across TCGA Database

**DOI:** 10.1155/2020/9143695

**Published:** 2020-11-03

**Authors:** Xin Zhao, Yu Tang, Haoyu Ren, Yi Lei

**Affiliations:** ^1^Department of Urology, The Affiliated Hospital of Southwest Medical University, Luzhou, China; ^2^Sichuan Clinical Research Center for Nephropathy, China; ^3^Department of Urology, The First Affiliated Hospital of Chongqing Medical University, Chongqing, China; ^4^Department of General, Visceral and Transplant Surgery, Ludwig-Maximilians-University, Munich, Germany; ^5^Department of Endocrinology and Metabolism, The Affiliated Hospital of Southwest Medical University, Luzhou, China; ^6^Cardiovascular and Metabolic Diseases Key Laboratory of Luzhou, China

## Abstract

**Background:**

Bladder cancer (BCa) is a common urothelial malignancy. The Cancer Genome Atlas (TCGA) database allows for an opportunity to analyze the relationship between gene expression and clinical outcomes in bladder cancer patients. This study is aimed at identifying prognosis-related genes in the bladder cancer microenvironment.

**Methods:**

Immune scores and stromal scores were calculated by applying the ESTIMATE algorithm. We divided bladder cancer patients into high and low groups based on their immune/stromal scores. Then, differentially expressed genes (DEGs) were identified in bladder cancer patients based on the TCGA database. We evaluated the correlation between immune/stromal scores and clinical characteristics as well as prognosis. Finally, we validated identified genes associated with bladder cancer prognosis through a cohort study in the Gene Expression Omnibus (GEO) database.

**Results:**

A higher stromal score was associated with female (vs. male*p* = 0.037), age > 65 (vs.age ≤ 65 *p* = 0.015), T3/4 (vs. T1/2,*p* < 0.001), N status(*p* = 0.016), and pathological high grade (vs. low grade*P* < 0.001). By analyzing DEGs, there were 1125 genes commonly upregulated, and 209 genes were commonly downregulated. Protein-protein interaction networks further showed the important protein that may be involved in the biological behavior and prognosis of BCa, such as FN1, CXCL12, CD3E, LCK, and ZAP70. A total of 14 DEGs were found to be associated with overall survival of bladder cancer. After validation by a cohort of 165 BCa cases with detailed follow-up information from GSE13507, 10 immune-associated DEGs were demonstrated to be predictive of prognosis in BCa. Among them, 5 genes have not been reported previously associated with the prognosis of BCa, including BTBD16, OLFML2B, PRRX1, SPINK4, and SPON2.

**Conclusions:**

Our study elucidated tight associations between stromal score and clinical characteristics as well as prognosis in BCa. Moreover, we obtained a group of genes closely related to the prognosis of BCa in the tumor microenvironment.

## 1. Introduction

Bladder cancer (BCa) is the fourth most common cancer in men and the twelfth in women and the leading cause of cancer-related mortality [1, 2]. Epidemiology studies reported that men have a four-fold higher incidence of bladder cancer than women [3]. Urothelial carcinoma accounts for 90% of BCa cases, whereas the remaining 10% cases are squamous cell carcinoma (SCC) or adenocarcinomas [4]. Bladder cancer treatment may include surgery, chemotherapy, radiotherapy, immunotherapy, and targeted therapy. Despite significant improvement in cancer treatment, two-thirds of patients with UBC will have a recurrence or disease progression within 5 years, leading to poor prognosis [5].

The tumor microenvironment consists of immune cells, mesenchymal cells, endothelial cells, along with inflammatory mediators, and extracellular matrix molecules [6]. Immune cells and stromal cells are the two most important nontumor cells in the tumor microenvironment. Previous evidences indicate that the components in the tumor microenvironment have an important role in promoting the proliferation and invasion of BC [7–9]. High stromal tumor-infiltrating lymphocytes within the tumor immune microenvironment is indicative of an inflamed subtype with an 80% 5-year disease-specific survival, and a lack of immune infiltrates is associated with an uninflamed subtype with a survival rate of smaller than 25%. A separate immune evading phenotype with upregulated immune checkpoints is associated with poor prognosis in bladder cancer [5]. Tumor microenvironment may have protective functions in other cancers, such as head and neck squamous cell carcinoma and glioblastoma [10, 11]. However, there are few reports that systematically investigate the genes involved in the tumor microenvironment and their associations with prognosis in bladder cancer.

To better understand the impact of tumor genetic composition on clinical outcomes, the Cancer Genome Atlas (TCGA), a comprehensive genome-wide gene expression collection, has been established to discover genomic abnormalities around the world [12]. Tomczak et al. designed an algorithm called ESTIMATE (Estimation of STromal and Immune cells in MAlignant Tumor tissues using Expression data), which calculated immune and stromal scores to predict the infiltration of nontumor cells by analyzing specific gene expression signature [13]. In this present study, we identified a list of tumor microenvironment-related genes, which are closely related to the prognosis in bladder cancer by using the TCGA database and ESTIMATE algorithm. Moreover, we validated prognosis-related genes in a different bladder cancer cohort available from the Gene Expression Omnibus (GEO) database.

## 2. Materials and Methods

### 2.1. Data Acquisition

Gene expression profile and clinical data including gender, age, clinical stage, overall survival for 412 patients with BCa, and 19 adjacent nontumor tissues were obtained from the TCGA data portal (https://tcga-data.nci.nih.gov/tcga/). The histological subtype of the bladder cancer is muscle-invasive urothelial carcinoma. Immune scores and stromal scores were calculated by the ESTIMATE algorithm [14]. For validation, the expression data and clinical information of 165 eligible samples were identified from the GEO database (GSE13507) (https://www.ncbi.nlm.nih.gov/gds). Only histologically verified transitional cell carcinoma samples were selected in the GEO dataset (GSE13507).

### 2.2. Differentially Expressed Gene Analysis

Differentially expressed genes (DEGs) were determined between 412 BCa, and 19 nontumor counterparts were performed using the package “limma” in R. Genes were considered as DEGs following the thresholds of ∣log2 fold change (FC) | >2 and adjusted *p* value < 0.05. Expression profiling of the DEGs was performed using the R package heatmap. The overlap of DEGs was determined using the R package Venn diagrams.

### 2.3. Functional Enrichment Analysis of DEGs

Functional enrichment analysis of DEGs was performed by R packages “clusterProfiler”, “http://org.Hs.eg.db”, “enrichplot”, and “ggplot2” to identify gene ontology (GO) categories, including biological processes (BP), cellular components (CC), or molecular functions (MF). The abovementioned packages were also used to perform KEGG (Kyoto Encyclopedia of Genes and Genomes) pathway enrichment analysis [15]. The protein-protein interaction (PPI) network was built by Search Tool for the Retrieval of Interacting Genes/Proteins (STRING) (https://string-db.org); interaction score > 0.95 was set as the cut off to screen for the key PPI nodes [16].

### 2.4. Overall Survival Analyses

The survival analysis was assessed by Kaplan-Meier methods. The patients with BCa were split into high-score group and low-score group according to the median immune/stromal scores. Overall survival curves were compared between the two groups using the Kaplan-Meier method. To investigate the associations of DEGs with overall survival, the Kaplan-Meier method was utilized to compare the survival rate difference between high and low groups stratified by the median gene expression levels. Log-rank tests were used to assess the statistical differences. Univariate and multivariate survival analyses were performed between overall survival, DEG expression levels, and clinical features, including age, gender, T clinical stage, N clinical stage, and M clinical stage and grade, using the Cox regression models of the R package survival. Hazard ratio (HR) and 95% confidence interval (CI) of HR were extracted from the Cox regression models. Prognosis-associated genes were classified into protective genes (0 < HR < 1) and risk genes (HR > 1) according to their HR values. *p* values < 0.05 were considered statistically significant.

### 2.5. Statistical Analysis

The correlations between clinicopathological parameters and immune/stromal scores were analyzed using a two-side student *t* test. All of the statistical tests were performed in R version 3.3 (https://www.r-project.org/). *p* values < 0.05 were considered as statistical significance.

## 3. Results

We downloaded gene expression profiles and clinicopathological parameters of 412 patients with BCa from the TCGA database. The total study population comprised 304 (73.8%) men and 108 (26.2%) women. Stromal scores ranged from -2537.32 to 2146.99, and immune scores ranged from -1591.88 to 3176.41.

### 3.1. The Association between Immune and Stromal Scores and Clinicopathological Parameters

**Figure 1:**
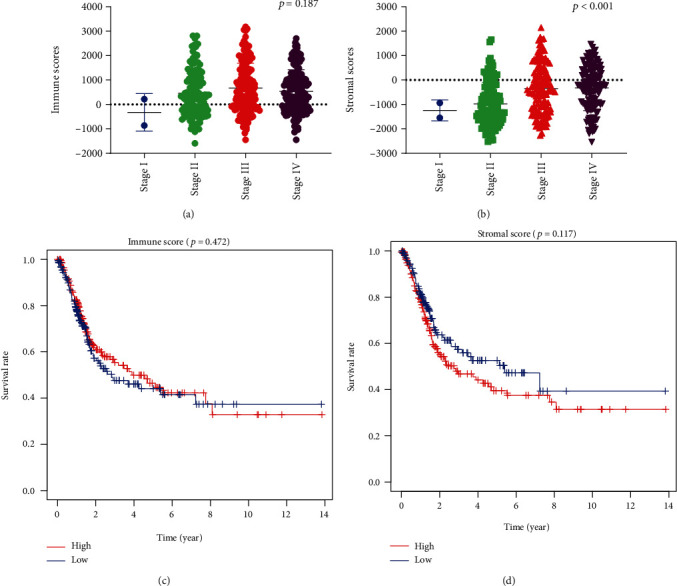
The association between immune and stromal scores and clinical stage. (a) Distribution of the immune scores of bladder cancer by clinical stage. There was no association between the clinical stage of bladder cancer and immune scores (*n* = 412, *p* = 0.187). (b) Distribution of the stromal scores of bladder cancer by clinical stage. There was a significant association between the clinical stage of bladder cancer and stromal scores (*n* = 412, *p* < 0.001). (c) According to the ranking of immune scores, bladder cancer cases were divided into low and high groups. (d) According to the ranking of stromal scores, bladder cancer cases were divided into low and high groups.

Based on the latest AJCC staging, there was no statistical difference between immune score and the clinical stage (*p* = 0.187) (Figure 1(a)). However, stromal cell scores were significantly associated with the clinical stage (*p* < 0.001) (Figure 1(b)). We divided the 412 bladder cancer cases into high and low score groups. Kaplan-Meier survival curves (Figure 1(c)) showed that the low stromal scores group had a lower mortality rate than the high scores group (33.5% and 45.3%, *p* = 0.117). Cases with low immune scores had a lower mortality rate compared to patients with high scores group (Figure 1(d), 38.9% and 39.4%, *p* = 0.472), although no statistical differences were found.

**Figure 2:**
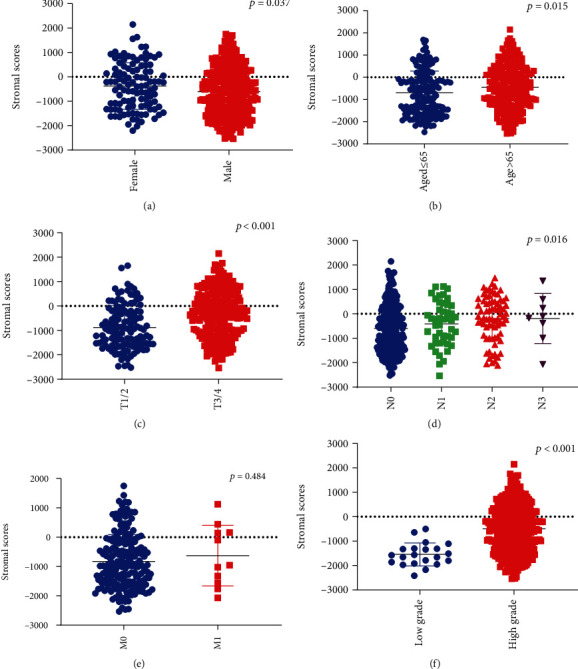
The association between stromal scores and clinicopathological parameters. (a) The scatter plot indicated that higher stromal scores were associated with female sex (vs. male sex, *p* = 0.037). (b) The scatter plot indicated that higher stromal scores were associated with age > 65 (vs. age ≤ 65, *p* = 0.015). (c) The scatter plot indicated that higher stromal scores were associated with T3/4 (vs. T1/2, *p* < 0.001). (d) The distribution of stromal scores stratified by N status. The scatter plot indicated that higher stromal scores were associated with higher N status (*p* = 0.016). (e) The scatter plot indicated no significant associations between stromal scores and M1 (vs. M0, *p* = 0.484). (f) The scatter plot indicated that higher stromal scores were associated with high grade (vs. low grade, *p* < 0.001).

To evaluate the correlations between clinicopathological parameters and immune/stromal scores, we compared and plotted the distribution of immune scores and stromal scores stratified by age, gender, T status, N status, M status, and Fuhrman grade. We found that higher stromal score was associated with female (vs. male, Figure 2(a)*p* = 0.037), age > 65 (vs. age ≤ 65, Figure 2(b),*p* = 0.015), T3/4 (vs. T1/2, Figure 2(c),*p* < 0.001), N status (Figure 2(d), *p* = 0.016), and pathological high grade (vs. low grade, Figure 2(f)*p* < 0.001), but was not associated with M status (Figure 2(e)*p* = 0.484). However, high immune scores were only associated with pathological high grade (vs. low grade, Supplementary Figure 1(f), *p* = 0.001), and no evidence supports the significant correlations between immune scores and age, gender, T status, N status, and M status (Supplementary Figure 1A-1E, *p* > 0.05).

### 3.2. Characteristics in Gene Heatmaps with Immune Scores and Stromal Scores in BCa

**Figure 3:**
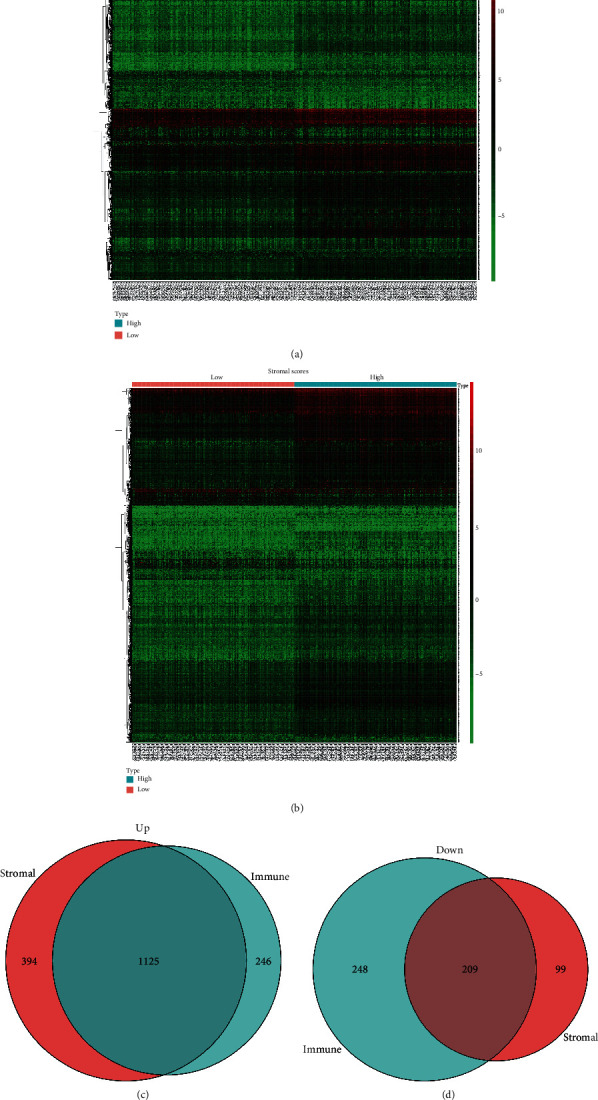
Heatmap and Venn diagram of the gene expression profiles with immune scores and stromal scores. In the heatmaps, red indicates that the gene is highly expressed, green indicates that the gene is lowly expressed, and black indicates genes with the same expression level. (a) Heatmap of the differentially expressed genes (DEGs) of immune scores (*p* < 0.05, fold change > 1.5). (b) Heatmap of the DEGs of stromal scores (*p* < 0.05, fold change > 1.5). (c) Venn diagrams showing the number of commonly upregulated DEGs in the stromal and immune score groups. (d) Downregulated DEGs.

Heatmaps showed the differential gene expression profiles based on the immune and stromal scores (Figures 3(a) and 3(b)). There were 1371 genes upregulated and 457 genes downregulated in the high immune scores group as compared to the low immune score group. A total of 1519 genes were upregulated, and 398 genes were downregulated in the high stromal score group as compared to the low stromal score group. Moreover, Venn diagrams (Figures 3(c) and 3(d)) showed that 1125 genes were commonly upregulated, and 209 genes were commonly downregulated.

### 3.3. Protein-Protein Interactions among Genes of Prognostic Value

**Figure 4:**
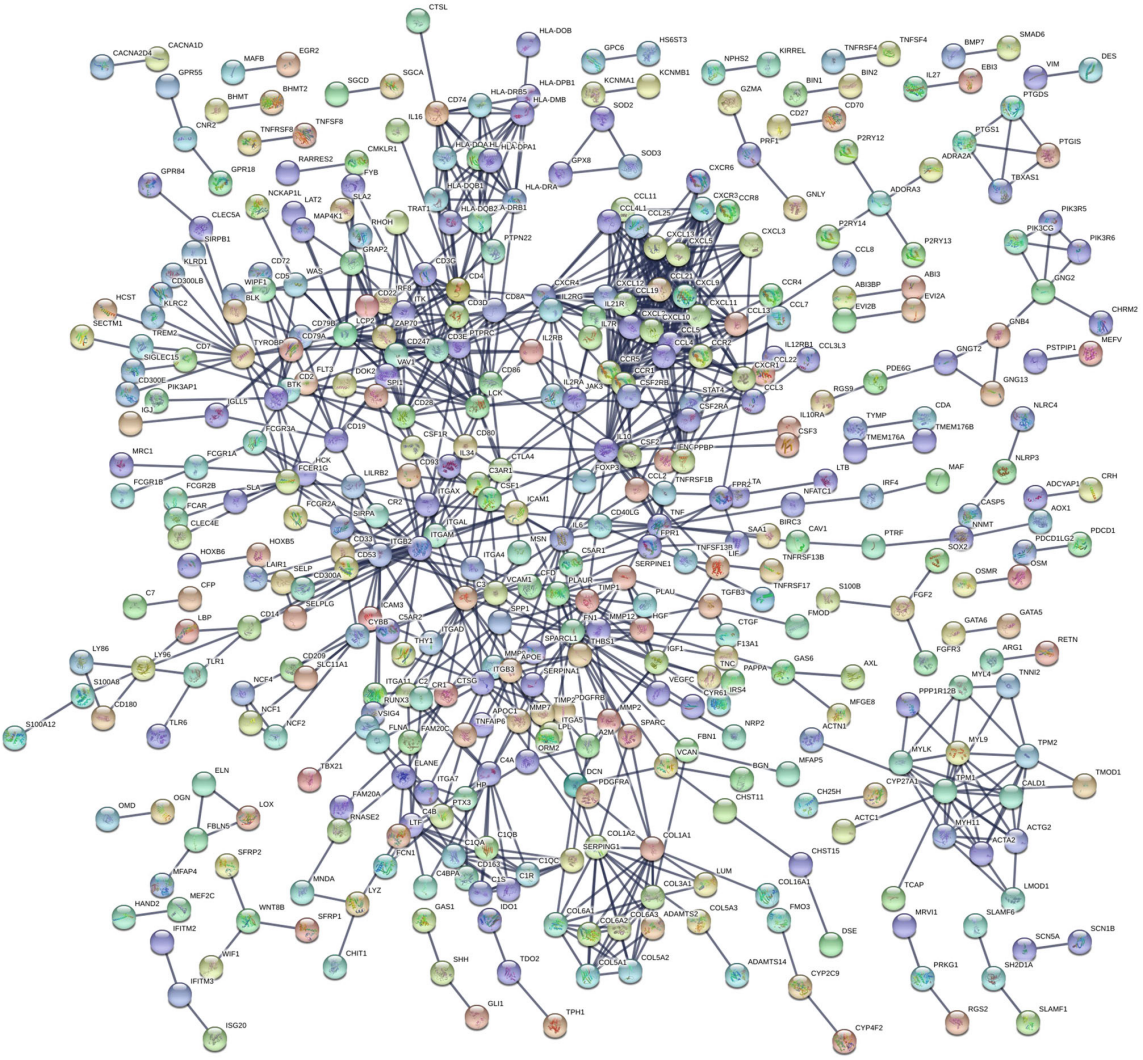
The PPI network. An interaction score of >0.95 was set as the cut off to screen the PPIs.

**Figure 5:**
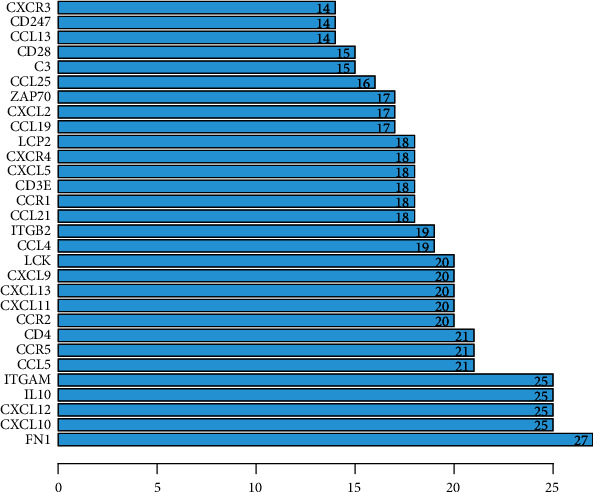
Important proteins in the PPI.

**Figure 6:**
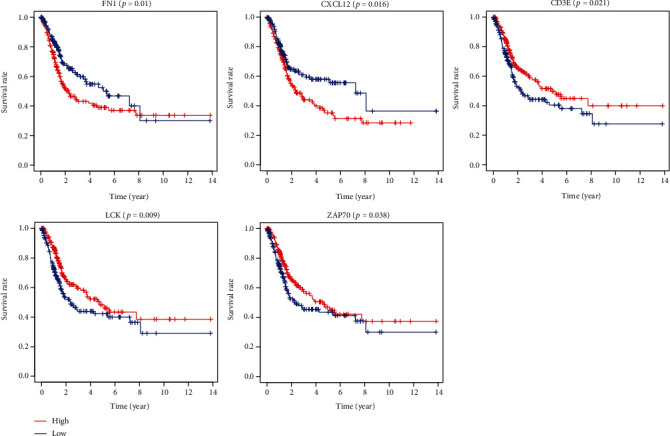
Correlation of key genes in the PPI network with overall survival in bladder cancer in the TCGA database. Comparison of DEGs with groups of high (red) and low (blue) expression. OS: overall survival.

We drew PPI networks by using the STRING (Figure 4). The number of protein nodes in the network diagram was shown in Figure 4. There were 13 genes in the network diagram with more than 20 interconnected nodes, such as FN1, CXCL10, CXCL12, IL10, ITGAM, CCL5, CCR5, CD4, CCR2, CXCL11, CXCL13, CXCL9, and LCK (Figure 5). It includes multiple genes that are closely related to immune response. Moreover, FN1, CXCL12, CD3E, LCK, and ZAP70 were key interconnected node genes in PPI networks and are also associated with overall survival in patients with bladder cancer (Figure 6).

### 3.4. GO and KEGG Enrichment Analysis of Genes

**Figure 7:**
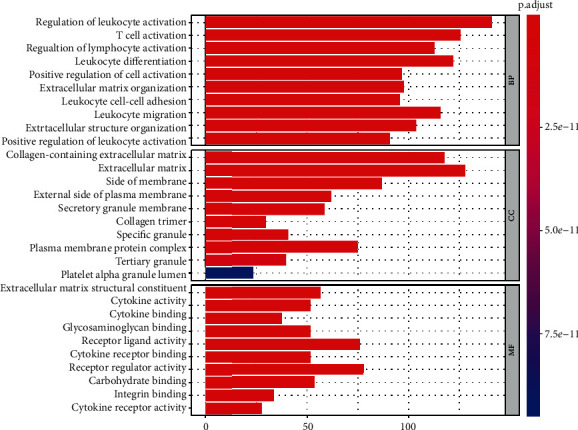
GO term analysis for DEGs significantly associated with overall survival in bladder cancer. BP: biological process. CC: cellular component. MF: molecular function.

**Figure 8:**
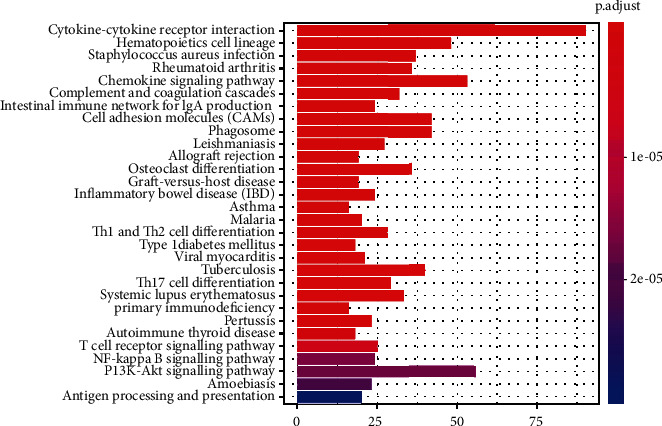
Bar plot for KEGG pathway analysis of DEGs significantly associated with overall survival.

Top GO terms included the regulation of leukocyte activation and T cell activation in biological processes, collagen-containing extracellular matrix and extracellular matrix in cellular components, and receptor-ligand activity and receptor regular activity in molecular functions (Figure 7). In addition, the results of KEGG pathway enrichment analysis of differentially expressed proteins are shown in Figure 8, and cytokine-cytokine receptor interaction, PI3K-Akt signaling pathway, and chemokine signaling pathway are the top three enrichment pathway for differentially expressed proteins.

### 3.5. Correlation of Expression of Individual DEGs in Overall Survival and Validation in the GEO Database

**Figure 9:**
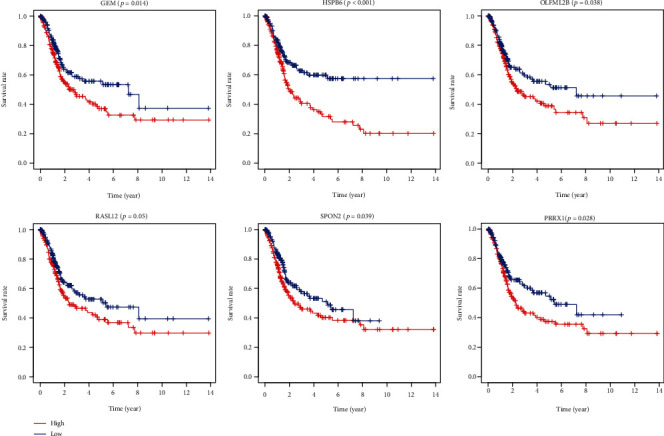
Correlation of individual DEG expression with overall survival in bladder cancer in the TCGA database. Comparison of DEGs with groups of high (red) and low (blue) expression. OS: overall survival.

**Figure 10:**
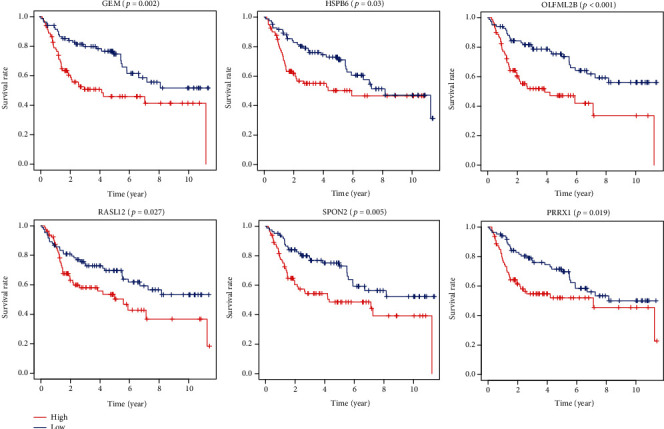
Validation of the correlation of differentially expressed genes with prognosis by the GEO database. Comparison of DEGs with groups of high (red) and low (blue) expression. OS: overall survival.

We generated Kaplan-Meier survival curves to evaluate the role of individual DEGs in overall survival in bladder cancer. A total of 274 DEGs were found to be associated with the prognosis of bladder cancer (*p* < 0.05, selected genes are shown in Figure 9). To further validate, the prognosis-related genes found in the TCGA database are significant in other bladder cancer cases. We analyzed a cohort study of 165 bladder cancer cases from the GEO database. A total of 29 genes were validated (Figure 10) to be significantly associated with bladder cancer prognosis (Table 1).

Univariate and multivariate analyses confirmed that 9 genes were risk genes and 5 protective genes in the TCGA cohort (*p* < 0.05 for all cases, Supplementary Table 1-2). Of 14 prognosis-associated genes, 5 risk genes (CALD1, TNC, OLFML2B, PRRX1, and SPON2) and 5 protective genes (HOXB3, HOXB6, MOGAT2, BTBD16, and SPINK4) were validated by univariate survival analysis in the GEO cohort (*p* < 0.05 for all cases, Supplementary Table 1-2). Among them, 5 genes have not been reported previously associated with the prognosis of BCa, including BTBD16, OLFML2B, PRRX1, SPINK4, and SPON2.

## 4. Discussion

In our study, we found that immune scores and stromal scores were associated with BCa patients' survival based on TCGA datasets, although no statistical differences were found in K-M survival analysis. Stromal scores were associated with multiple clinicopathological parameters, including AJCC stage, age, gender, T status, N status, and Fuhrman grade of BCa. However, immune scores were only associated with Fuhrman grade, and no significant correlation was found between immune scores and AJCC stage, age, gender, T status, N status, and M status. These results indicated that stromal cells in the tumor microenvironment may play a more important role in multiple biological behaviors, affecting the occurrence, development, and prognosis of bladder cancer, compared with immune cells.

We further performed heatmaps to show the differential gene expression profiles according to the immune and stromal scores. In addition, Venn diagrams showed that 1125 DEGs were commonly upregulated, and 209 DEGs were commonly downregulated. Moreover, protein-protein interaction networks were constructed based on commonly differentially expressed genes. There are many key proteins in the PPI networks involved in immune/inflammation response, such as IL10 and CD4. Luo reported that blocking IL-10 can enhance the effect of BCG immunotherapy for bladder cancer [17]. Previous research found that CD3D/CD4 ratio is an important marker for the prognosis of bladder cancer [18].

The results of GO enrichment analysis suggested that many of the enriched differential genes are related to immune cell activity (leukocyte activation and T cell activation) and stromal cell components (collagen-containing extracellular matrix and extracellular matrix), indicating immune cells and stromal cells, as key components in the tumor microenvironment, may be involved in the development of bladder cancers. He et al. reported that inflammatory response-related KEGG pathway was significantly enriched in shOIP5 bladder cancer cell lines, including cytokine-cytokine receptor interaction and chemokine signaling pathway, which was in accordance with our present study [19]. The PI3K-Akt signaling pathway is a classic pathway for cell proliferation, thus playing an important role in the pathogenesis of multiple cancers. Previous researches have shown many oncogenes promote proliferation via the PI3K-Akt signaling pathway in bladder cancer [20, 21]. All these suggest that our analysis results are helpful to clarify the pathogenesis and mechanism of bladder cancer and provide new research ideas for the treatment of bladder cancer.

In recent years, increasingly more mRNAs have the potential to be molecular biomarkers for evaluating and predicting the prognosis in various cancer types [22–24]. However, there are few reports on the tumor microenvironment-related genes to predict cancer outcomes in bladder cancer. In our study, a total of 274 genes are significantly associated with overall survival in bladder cancer. Among the 274 genes, the proteins encoded by FN1, CXCL12, CD3E, LCK, and ZAP70 genes were also key proteins in PPI. FN1 has the largest number of interconnected nodes in the PPI, suggesting that it may be involved in multiple aspects of bladder cancer development. FN1 encodes fibronectin, a glycoprotein present in a soluble dimeric form in plasma, and itself is a potential urine biomarker for bladder cancer detection [20]. CXC chemokine ligand 12 (CXCL12) is an important member of the CXC subfamily of chemokines. Nazari et al. suggested that elevated protein and mRNA levels of CXCL12 are found in human bladder cancer, which plays a role during the genesis of BCa and its further development [25]. Punt et al. found that high CD3E expression was correlated with improved disease-specific survival in squamous cervical cancer [26]. Previous study lymphocyte-specific protein tyrosine kinase (Lck) was one of the key molecules regulating T-cell functions, which emerged as a novel druggable target molecule for the treatment of cancers [27]. Fu et al. demonstrated that ZAP70 (a tyrosine kinase of the Syk family) may play an important role in the T-cell receptor (TCR) signaling pathway, which facilitated PCa cell migration and invasion [28]. To our delight, the function of CD3E, Lck, and ZAP70 gene have not been reported in bladder cancer, indicating that functional studies on these genes may help us to more accurately understand the prognosis-related biological behavior of bladder cancers. Furthermore, they also provide a new idea for further exploring the molecular mechanism and targeted therapy of bladder cancer.

Importantly, we validated these OS-related genes in an independent cohort of 165 bladder cancer patients from the GEO database (GSE13507). A total of 10 genes were identified to be correlated with overall survival. Among these identified genes, 5 genes (CALD1, HOXB3, HOXB6, MOGAT2, and TNC) have been reported to be associated with the development or prognosis of bladder cancer, indicating that the results in our study are consistent with previous publications in bladder cancer [29–31]. The remaining 5 genes have not been found to be associated with the prognosis of bladder cancer (BTBD16, OLFML2B, PRRX1, SPINK4, and SPON2). Further research that focuses on these potential prognostic-related genes may find new biomarkers of bladder cancer. Meanwhile, these genes may lead to gene-mediated molecular targeting therapy.

## 5. Conclusions

In summary, high stromal is associated with females, age above 65, clinical T stage 3/4, clinical N status, and pathological high grade. We found 1125 differentially expressed genes associated with tumor microenvironment in bladder cancer. Ten genes were closely related to prognosis, and they may have the potential to become prognostic biomarkers for bladder cancer.

## Figures and Tables

**Table 1:** Genes significantly associated with overall survival of bladder cancer identified in both TCGA and GEO database.

Categories	Gene symbols
Genes have been reported to be associated with prognosis of the bladder cancer	ACOXL, ADAM12, CALD1, CD3G, CPXM2, CYR61, DGKI, GPR174, HOXB3, HOXB6, MFGE8, MOGAT2, PALLD, PTGFR, SLC26A5, TAGLN, TGFB1I1, TNC
Genes have not been reported for their prognostic value of the bladder cancer	ABCC9, ADAMTS16, BTBD16, GEM, HSPB6, OLFML2B, PRRX1, RASL12, SPINK4, SPON2, STK32A

## Data Availability

The datasets supporting the conclusions of this article are available in the Cancer Genome Atlas (TCGA) database and Gene Expression Omnibus (GEO) with the accession numbers GSE13507. All of those studies previously were approved by their respective institutional review boards.

## Abbreviations

BCa: Bladder cancer
TCGA: The Cancer Genome Atlas
GEO: Gene Expression Omnibus
DEGs: Differentially expressed genes
GO: Gene ontology
PPI: Protein–protein interaction
KEGG: Kyoto Encyclopedia of Genes and Genomes
STRING: Search Tool for the Retrieval of Interacting Genes/Proteins.

## Supplementary Materials

Supplementary 1 Supplementary Figure 1: the association between immune scores and clinicopathological parameters. (a) The scatter plot indicated that higher immune scores were associated with female sex (vs. male sex, *p* = 0.07). (b) The scatter plot indicated that higher immune scores were associated with age > 65 (vs. age ≤ 65, *p* = 0.707). (c) The scatter plot indicated that higher immune scores were associated with T3/4 (vs. T1/2, *p* = 0.090). (d) The distribution of immune scores stratified by N status. The scatter plot indicated that higher immune scores were associated with higher N status (*p* = 0.937). (e) The scatter plot indicated no significant associations between immune scores and M1 (vs. M0, *p* = 0.056). (f) The scatter plot indicated that higher immune scores were associated with high grade (vs. low grade, *p* = 0.001)

Supplementary 2 Supplementary Table 1: survival analyses between patients' overall survival and 14 gene expression levels in the TCGA dataset. Supplementary Table 2: survival analyses between patients' overall survival and 14 gene expression levels in the GEO dataset.

## References

[1] Siegel R. L., Miller K. D., Jemal A. (2018). Cancer statistics, 2018. *CA: A Cancer Journal for Clinicians*.

[2] Ferlay J., Soerjomataram I., Dikshit R. (2015). Cancer incidence and mortality worldwide: sources, methods and major patterns in GLOBOCAN 2012. *International Journal of Cancer*.

[3] Cook M. B., Dawsey S. M., Freedman N. D. (2009). Sex disparities in cancer incidence by period and age. *Cancer Epidemiology, Biomarkers & Prevention*.

[4] Salem H. K., Mahfouz S. (2012). Changing patterns (age, incidence, and pathologic types) of schistosoma-associated bladder cancer in Egypt in the past decade. *Urology*.

[5] Pfannstiel C., Strissel P. L., Chiappinelli K. B. (2019). The tumor immune microenvironment drives a prognostic relevance that correlates with bladder cancer subtypes. *Cancer Immunology Research*.

[6] Hanahan D., Weinberg R. A. (2000). The hallmarks of cancer. *Cell*.

[7] Muller K., Ellenberger C., Hoppen H. O., Schoon H. A. (2012). Immunohistochemical study of angiogenesis and angiogenic factors in equine granulosa cell tumours. *Research in Veterinary Science*.

[8] Eruslanov E., Neuberger M., Daurkin I. (2012). Circulating and tumor-infiltrating myeloid cell subsets in patients with bladder cancer. *International Journal of Cancer*.

[9] Said N., Theodorescu D. (2014). RhoGDI2 suppresses bladder cancer metastasis via reduction of inflammation in the tumor microenvironment. *OncoImmunology*.

[10] Curry J. M., Sprandio J., Cognetti D. (2014). Tumor microenvironment in head and neck squamous cell carcinoma. *Seminars in Oncology*.

[11] Cooper L. A., Gutman D. A., Chisolm C. (2012). The tumor microenvironment strongly impacts master transcriptional regulators and gene expression class of glioblastoma. *The American Journal of Pathology*.

[12] Hanahan D., Coussens L. M. (2012). Accessories to the crime: functions of cells recruited to the tumor microenvironment. *Cancer Cell*.

[13] Tomczak K., Czerwińska P., Wiznerowicz M. (2015). Review The Cancer Genome Atlas (TCGA): an immeasurable source of knowledge. *Contemporary oncology (Poznan, Poland)*.

[14] Yoshihara K., Shahmoradgoli M., Martínez E. (2013). Inferring tumour purity and stromal and immune cell admixture from expression data. *Nature Communications*.

[15] Huang D. W., Sherman B. T., Lempicki R. A. (2009). Systematic and integrative analysis of large gene lists using DAVID bioinformatics resources. *Nature Protocols*.

[16] Szklarczyk D., Franceschini A., Wyder S. (2015). STRING v10: protein-protein interaction networks, integrated over the tree of life. *Nucleic Acids Research*.

[17] Luo Y. (2014). Blocking IL-10 enhances bacillus Calmette-Guérin induced T helper type 1 immune responses and anti-bladder cancer immunity. *OncoImmunology*.

[18] Shi M. J., Meng X. Y., Wu Q. J., Zhou X. H. (2019). High CD3D/CD4 ratio predicts better survival in muscle-invasive bladder cancer. *Cancer Management and Research*.

[19] He X., Ding X., Wen D., Hou J., Ping J., He J. (2017). Exploration of the pathways and interaction network involved in bladder cancer cell line with knockdown of Opa interacting protein 5. *Pathology-Research and Practice*.

[20] Sathe A., Nawroth R. (2018). Targeting the PI3K/AKT/mTOR pathway in bladder cancer. *Methods in Molecular Biology*.

[21] Li Y., Guo G., Song J. (2017). B7-H3 promotes the migration and invasion of human bladder cancer cells via the PI3K/Akt/STAT3 signaling pathway. *Journal of Cancer*.

[22] Shahid M., Choi T. G., Nguyen M. N. (2016). An 8-gene signature for prediction of prognosis and chemoresponse in non-small cell lung cancer. *Oncotarget*.

[23] Nguyen M. N., Choi T. G., Nguyen D. T. (2015). CRC-113 gene expression signature for predicting prognosis in patients with colorectal cancer. *Oncotarget*.

[24] Shahid M., Cho K. M., Nguyen M. N. (2016). Prognostic value and their clinical implication of 89-gene signature in glioma. *Oncotarget*.

[25] Nazari A., Khorramdelazad H., Hassanshahi G. (2017). Biological/pathological functions of the CXCL12/CXCR4/CXCR7 axes in the pathogenesis of bladder cancer. *International Journal of Clinical Oncology*.

[26] Punt S., Houwing-Duistermaat J. J., Schulkens I. A. (2015). Correlations between immune response and vascularization qRT-PCR gene expression clusters in squamous cervical cancer. *Molecular Cancer*.

[27] Marhall A., Kazi J. U., Ronnstrand L. (2017). The Src family kinase LCK cooperates with oncogenic FLT3/ITD in cellular transformation. *Scientific Reports*.

[28] Fu D., Liu B., Zang L. E., Jiang H. (2016). MiR-631/ZAP70: a novel axis in the migration and invasion of prostate cancer cells. *Biochemical and Biophysical Research Communications*.

[29] Frohlich C., Albrechtsen R., Dyrskjot L., Rudkjaer L., Orntoft T. F., Wewer U. M. (2006). Molecular profiling of ADAM12 in human bladder cancer. *Clinical Cancer Research*.

[30] Liu Y., Wu X., Wang G., Hu S., Zhang Y., Zhao S. (2019). CALD1, CNN1, and TAGLN identified as potential prognostic molecular markers of bladder cancer by bioinformatics analysis. *Medicine (Baltimore)*.

[31] Masson-Lecomte A., Lopez de Maturana E., Goddard M. E. (2016). Inflammatory-related genetic variants in non-muscle-invasive bladder cancer prognosis: a multimarker Bayesian assessment. *Cancer Epidemiology, Biomarkers & Prevention*.

