# Supplementary figures and images for: Identification of Prognosis-Related Genes in Bladder Cancer Microenvironment across TCGA Database
Source: Biomed Res Int. 2020 Nov 3;2020:9143695. doi: 10.1155/2020/9143695 (PMC7658688; doi:10.1155/2020/9143695)

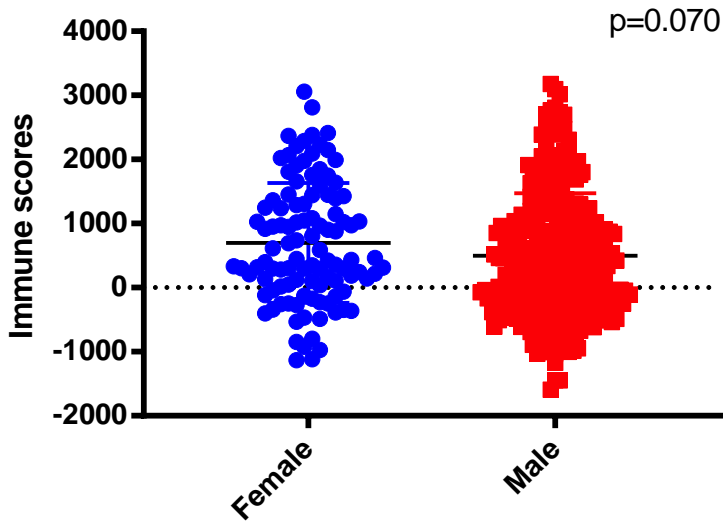

Supplement: Supplementary 1 — Supplementary Figure 1: the association between immune scores and clinicopathological parameters. (a) The scatter plot indicated that higher immune scores were associated with female sex (vs. male sex, p = 0.07). (b) The scatter plot indicated that higher immune scores were associated with age > 65 (vs. age ≤ 65, p = 0.707). (c) The scatter plot indicated that higher immune scores were associated with T3/4 (vs. T1/2, p = 0.090). (d) The distribution of immune scores stratified by N status. The scatter plot indicated that higher immune scores were associated with higher N status (p = 0.937). (e) The scatter plot indicated no significant associations between immune scores and M1 (vs. M0, p = 0.056). (f) The scatter plot indicated that higher immune scores were associated with high grade (vs. low grade, p = 0.001) [file 9143695.f1.zip › Supplementary Figure1A.pdf]

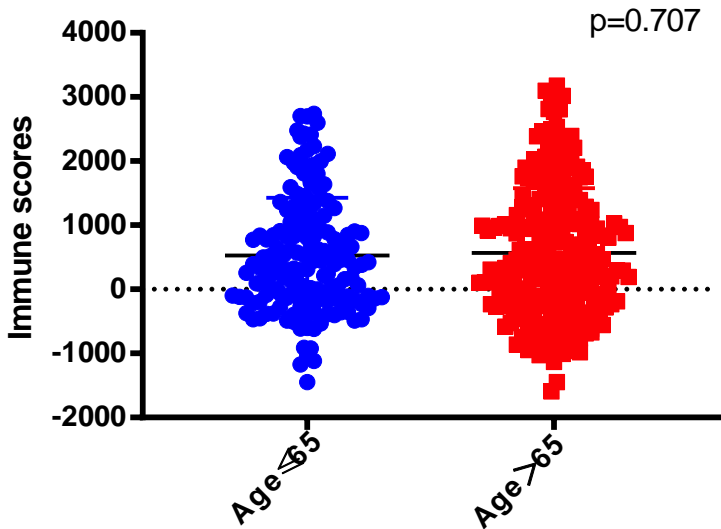

Supplement: Supplementary 1 — Supplementary Figure 1: the association between immune scores and clinicopathological parameters. (a) The scatter plot indicated that higher immune scores were associated with female sex (vs. male sex, p = 0.07). (b) The scatter plot indicated that higher immune scores were associated with age > 65 (vs. age ≤ 65, p = 0.707). (c) The scatter plot indicated that higher immune scores were associated with T3/4 (vs. T1/2, p = 0.090). (d) The distribution of immune scores stratified by N status. The scatter plot indicated that higher immune scores were associated with higher N status (p = 0.937). (e) The scatter plot indicated no significant associations between immune scores and M1 (vs. M0, p = 0.056). (f) The scatter plot indicated that higher immune scores were associated with high grade (vs. low grade, p = 0.001) [file 9143695.f1.zip › Supplementary Figure1B.pdf]

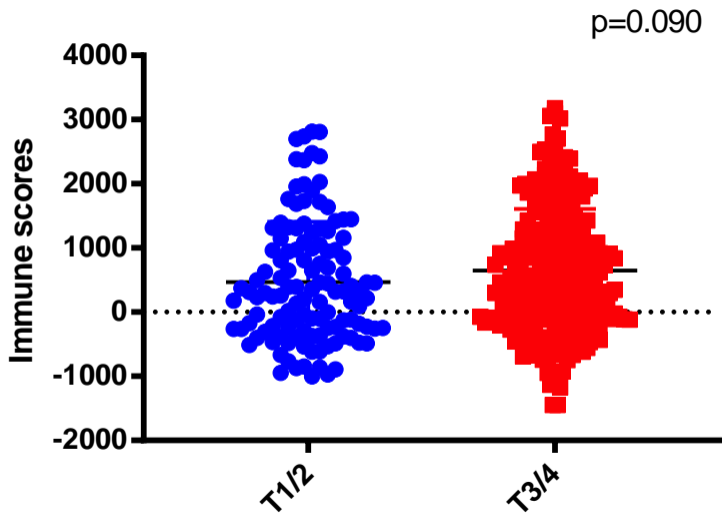

Supplement: Supplementary 1 — Supplementary Figure 1: the association between immune scores and clinicopathological parameters. (a) The scatter plot indicated that higher immune scores were associated with female sex (vs. male sex, p = 0.07). (b) The scatter plot indicated that higher immune scores were associated with age > 65 (vs. age ≤ 65, p = 0.707). (c) The scatter plot indicated that higher immune scores were associated with T3/4 (vs. T1/2, p = 0.090). (d) The distribution of immune scores stratified by N status. The scatter plot indicated that higher immune scores were associated with higher N status (p = 0.937). (e) The scatter plot indicated no significant associations between immune scores and M1 (vs. M0, p = 0.056). (f) The scatter plot indicated that higher immune scores were associated with high grade (vs. low grade, p = 0.001) [file 9143695.f1.zip › Supplementary Figure1C.pdf]

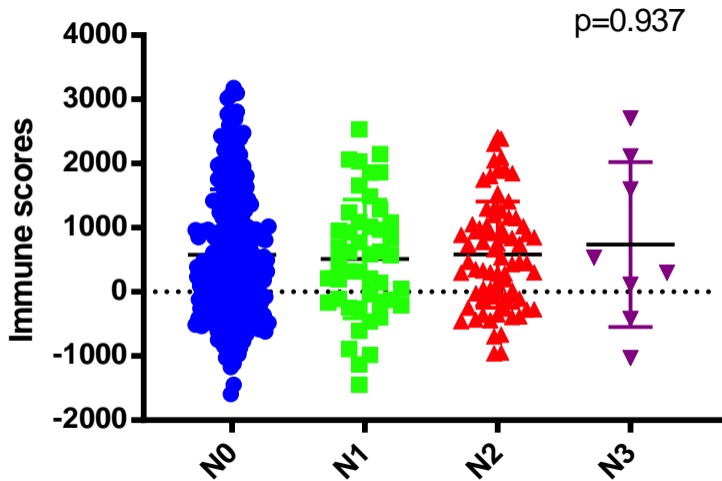

Supplement: Supplementary 1 — Supplementary Figure 1: the association between immune scores and clinicopathological parameters. (a) The scatter plot indicated that higher immune scores were associated with female sex (vs. male sex, p = 0.07). (b) The scatter plot indicated that higher immune scores were associated with age > 65 (vs. age ≤ 65, p = 0.707). (c) The scatter plot indicated that higher immune scores were associated with T3/4 (vs. T1/2, p = 0.090). (d) The distribution of immune scores stratified by N status. The scatter plot indicated that higher immune scores were associated with higher N status (p = 0.937). (e) The scatter plot indicated no significant associations between immune scores and M1 (vs. M0, p = 0.056). (f) The scatter plot indicated that higher immune scores were associated with high grade (vs. low grade, p = 0.001) [file 9143695.f1.zip › Supplementary Figure1D.pdf]

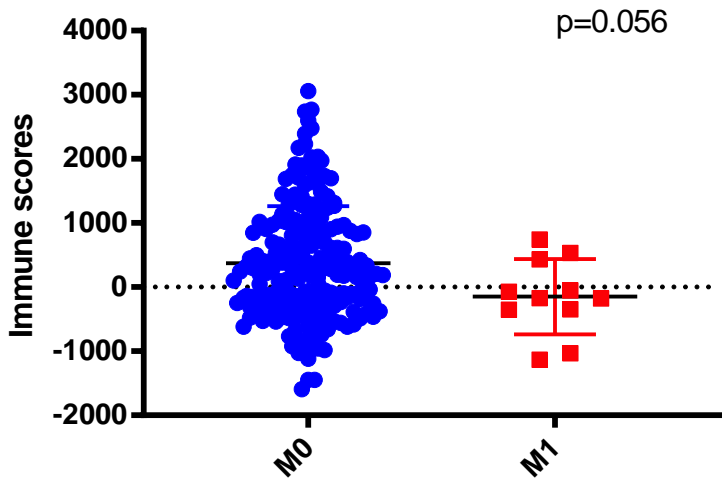

Supplement: Supplementary 1 — Supplementary Figure 1: the association between immune scores and clinicopathological parameters. (a) The scatter plot indicated that higher immune scores were associated with female sex (vs. male sex, p = 0.07). (b) The scatter plot indicated that higher immune scores were associated with age > 65 (vs. age ≤ 65, p = 0.707). (c) The scatter plot indicated that higher immune scores were associated with T3/4 (vs. T1/2, p = 0.090). (d) The distribution of immune scores stratified by N status. The scatter plot indicated that higher immune scores were associated with higher N status (p = 0.937). (e) The scatter plot indicated no significant associations between immune scores and M1 (vs. M0, p = 0.056). (f) The scatter plot indicated that higher immune scores were associated with high grade (vs. low grade, p = 0.001) [file 9143695.f1.zip › Supplementary Figure1E.pdf]

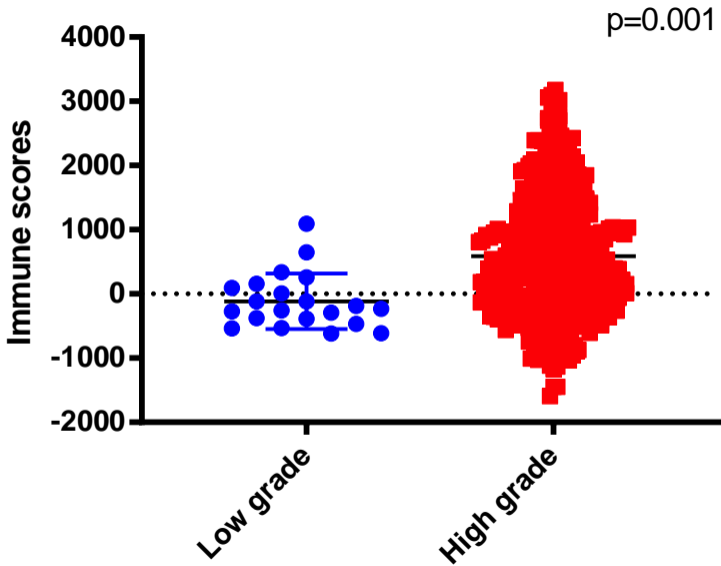

Supplement: Supplementary 1 — Supplementary Figure 1: the association between immune scores and clinicopathological parameters. (a) The scatter plot indicated that higher immune scores were associated with female sex (vs. male sex, p = 0.07). (b) The scatter plot indicated that higher immune scores were associated with age > 65 (vs. age ≤ 65, p = 0.707). (c) The scatter plot indicated that higher immune scores were associated with T3/4 (vs. T1/2, p = 0.090). (d) The distribution of immune scores stratified by N status. The scatter plot indicated that higher immune scores were associated with higher N status (p = 0.937). (e) The scatter plot indicated no significant associations between immune scores and M1 (vs. M0, p = 0.056). (f) The scatter plot indicated that higher immune scores were associated with high grade (vs. low grade, p = 0.001) [file 9143695.f1.zip › Supplementary Figure1F.pdf]
