# Supplementary material for: Identification of Prognosis-Related Genes in Bladder Cancer Microenvironment across TCGA Database
Source: Biomed Res Int. 2020 Nov 3;2020:9143695. doi: 10.1155/2020/9143695 (PMC7658688; doi:10.1155/2020/9143695)
Supplement: Supplementary 2 — Supplementary Table 1: survival analyses between patients' overall survival and 14 gene expression levels in the TCGA dataset. Supplementary Table 2: survival analyses between patients' overall survival and 14 gene expression levels in the GEO dataset. [file 9143695.f2.docx]

Supplementary Table1. survival analyses between patients’ overall survival and *14* gene expression levels in the TCGA dataset

|  |  | Univariate survival alysis | | | | Multivariate survival alysis | | | |
| --- | --- | --- | --- | --- | --- | --- | --- | --- | --- |
|  | Cutoff | OR | 2.5%CI | 97.5%CI | P value | OR | 2.5%CI | 97.5%CI | P value |
| CALD1 | 15.44 | 1.60 | 1.16 | 2.21 | 0.00 | 2.14 | 1.22 | 3.77 | 0.01 |
| DGKI | 0.07 | 1.53 | 1.11 | 2.11 | 0.01 | 1.76 | 1.02 | 3.05 | 0.04 |
| HOXB3 | 2.47 | 0.52 | 0.38 | 0.72 | 0.00 | 0.44 | 0.25 | 0.78 | 0.00 |
| HOXB6 | 2.36 | 0.69 | 0.50 | 0.95 | 0.02 | 0.53 | 0.30 | 0.95 | 0.03 |
| MOGAT2 | 0.13 | 0.69 | 0.50 | 0.95 | 0.02 | 0.57 | 0.32 | 0.99 | 0.05 |
| PALLD | 10.13 | 1.51 | 1.09 | 2.08 | 0.01 | 2.08 | 1.18 | 3.69 | 0.01 |
| TNC | 5.41 | 1.41 | 1.02 | 1.93 | 0.03 | 2.41 | 1.40 | 4.16 | 0.00 |
| ABCC9 | 0.21 | 1.72 | 1.25 | 2.38 | 0.00 | 1.98 | 1.10 | 3.56 | 0.02 |
| ADAMTS16 | 0.21 | 1.47 | 1.06 | 2.02 | 0.02 | 2.71 | 1.51 | 4.85 | 0.00 |
| BTBD16 | 2.31 | 0.68 | 0.49 | 0.93 | 0.02 | 0.45 | 0.26 | 0.78 | 0.00 |
| OLFML2B | 5.50 | 1.40 | 1.02 | 1.93 | 0.04 | 2.35 | 1.31 | 4.23 | 0.00 |
| PRRX1 | 1.72 | 1.43 | 1.04 | 1.97 | 0.03 | 1.79 | 1.02 | 3.14 | 0.04 |
| SPINK4 | 0.17 | 0.66 | 0.48 | 0.91 | 0.01 | 0.41 | 0.24 | 0.71 | 0.00 |
| SPON2 | 5.29 | 1.40 | 1.02 | 1.92 | 0.04 | 1.78 | 1.02 | 3.10 | 0.04 |

Supplementary Table2. survival analyses between patients’ overall survival and *14* gene expression levels in the GEO dataset

|  |  | Univariate survival alysis | | | | Multivariate survival alysis | | | |
| --- | --- | --- | --- | --- | --- | --- | --- | --- | --- |
|  | Median | OR | 2.5%CI | 97.5%CI | P value | OR | 2.5%CI | 97.5%CI | P value |
| CALD1 | 9.61 | 1.75 | 1.04 | 2.94 | 0.03 | 1.59 | 0.65 | 3.92 | 0.31 |
| DGKI | 6.92 | 0.58 | 0.34 | 0.98 | 0.04 | 0.97 | 0.39 | 2.44 | 0.95 |
| HOXB3 | 8.69 | 0.58 | 0.35 | 0.98 | 0.04 | 0.97 | 0.34 | 2.80 | 0.96 |
| HOXB6 | 8.03 | 0.51 | 0.30 | 0.86 | 0.01 | 0.89 | 0.29 | 2.74 | 0.85 |
| MOGAT2 | 7.31 | 0.46 | 0.27 | 0.79 | 0.00 | 0.71 | 0.29 | 1.79 | 0.47 |
| PALLD | 10.12 | 1.61 | 0.96 | 2.69 | 0.07 | 1.57 | 0.63 | 3.90 | 0.33 |
| TNC | 7.95 | 2.04 | 1.20 | 3.45 | 0.01 | 1.65 | 0.63 | 4.31 | 0.30 |
| ABCC9 | 7.16 | 0.52 | 0.31 | 0.89 | 0.02 | 1.42 | 0.59 | 3.41 | 0.44 |
| ADAMTS16 | 7.22 | 0.55 | 0.32 | 0.93 | 0.03 | 0.51 | 0.21 | 1.24 | 0.14 |
| BTBD16 | 12.41 | 0.57 | 0.34 | 0.95 | 0.03 | 1.33 | 0.42 | 4.20 | 0.63 |
| OLFML2B | 7.87 | 2.49 | 1.45 | 4.29 | 0.00 | 0.87 | 0.27 | 2.82 | 0.81 |
| PRRX1 | 7.58 | 1.97 | 1.16 | 3.33 | 0.01 | 0.67 | 0.22 | 2.02 | 0.48 |
| SPINK4 | 7.48 | 0.56 | 0.33 | 0.94 | 0.03 | 0.76 | 0.31 | 1.87 | 0.55 |
| SPON2 | 7.41 | 2.15 | 1.27 | 3.64 | 0.00 | 1.03 | 0.38 | 2.80 | 0.95 |
